# Perioperative Marinobufagenin (MBG) Measurement May Improve Acute Kidney Injury Risk Assessment in Patients Undergoing Major Cardiac Surgery: A Proof-of-Concept Study

**DOI:** 10.3390/medicina60071079

**Published:** 2024-06-30

**Authors:** Davide Bolignano, Giuseppe Filiberto Serraino, Patrizia Pizzini, Federica Jiritano, Mariateresa Zicarelli, Belinda Spoto, Marco Mobrici, Michela Musolino, Désirée Napolitano, Alessandra Testa, Michele Andreucci, Pasquale Mastroroberto, Giuseppe Coppolino

**Affiliations:** 1 Department of Medical and Surgical Sciences, University “Magna Graecia” of Catanzaro, 88100 Catanzaro, Italy; 2 Department of Experimental and Clinical Medicine, University “Magna Graecia” of Catanzaro, 88100 Catanzaro, Italy; 3 Italian National Council of Research (CNR)–Institute of Clinical Physiology, 89100 Reggio Calabria, Italy; 4 Department of Health Sciences, University “Magna Graecia” of Catanzaro, 88100 Catanzaro, Italy

**Keywords:** cardiac surgery, acute kidney injury, marinobufagenin, STS-AKI score, biomarker

## Abstract

*Background and Objectives:* Acute kidney injury (AKI) remains a significant complication following major cardiac surgery. Marinobufagenin (MBG), a cardiotonic steroid involved in sodium balance and blood pressure regulation, has been linked to organ damage after ischemia–reperfusion events. This pilot, prospective study investigates the utility of circulating MBG to improve AKI risk assessment in cardiac surgery patients as a stand-alone biomarker and after inclusion in a validated risk model (STS-AKI score). *Materials and Methods:* We included 45 patients undergoing elective cardiac surgery. The MBG levels were measured preoperatively and at 4, 8, and 12 h post-surgery. The AKI was defined according to the KDIGO guidelines. Statistical analyses assessed the diagnostic and prognostic utility of MBG and its integration with the STS-AKI score. *Results:* An AKI occurred in 26.7% of the patients. The STS-AKI score performed well in this cohort (AUC: 0.736). The MBG levels displayed a decreasing trend in the whole population after surgery (*p* = 0.02). However, in the AKI patients, MBG increased at 4 and 8 h before decreasing at 12 h post-surgery. The MBG changes from the baseline to 8 h and from 8 to 12 h post-surgery showed a remarkable diagnostic accuracy for an AKI (AUCs: 0.917 and 0.843, respectively). Integrating these MBG changes with the STS-AKI score significantly improved the model performance, including discrimination, calibration, and risk reclassification. *Conclusions*: The MBG measurement, particularly any dynamic changes post-surgery, enhances AKI risk stratification in cardiac surgery patients. Integrating MBG with the STS-AKI score offers more accurate risk predictions, potentially leading to better patient management and outcomes.

## 1. Introduction

Despite advancements in surgical techniques and perioperative care, the incidence of acute kidney injury (AKI) remains alarmingly high in patients undergoing major cardiac surgery [1].

An AKI has a significant impact on patient morbidity and mortality, increases the length of a hospital stay, and may elicit permanent renal failure [2]. Determining the optimal preoperatory risk stratification is thus mandatory for preventing complications and predisposing timely interventions.

The Society of Thoracic Surgeons (STS) AKI score is one of the most employed risk prediction tools for a postoperative AKI after cardiac surgery [3]. The key factors included in this score are patient demographics, comorbid conditions, preoperative renal function, type and urgency of the surgery, and intraoperative parameters such as cardiopulmonary bypass time.

One of the primary strengths of the STS-AKI score is its derivation from a large, multicenter database, which enhances its generalizability and robustness [4]. Additionally, unlike other tools specifically focusing on a severe AKI that requires dialysis (e.g., the Cleveland Clinic or the SRI scores) [5], the STS score categorizes the AKI risk into several levels, facilitating clinical decision-making and patient counseling. However, despite its widespread use, this score may have sub-optimal predictive accuracy in individuals with conserved preoperative renal function and its calibration may vary across different populations and surgical settings [6]. The inclusion of additional AKI biomarkers to this score could help improve the overall model performance, eventually leading to a more accurate risk stratification [7,8].

Marinobufagenin (MBG), an endogenous cardiotonic steroid (ECS) involved in the regulation of the sodium balance and blood pressure, has recently been implicated in the pathophysiology of organ damage following ischemia–reperfusion [9,10]. Altered levels of MBG have been associated with adverse outcomes in various cardiovascular conditions and may reflect disease severity in renal patients [11,12]. Nevertheless, its prognostic role with respect to a clinical AKI remains, to date, unexplored.

Given these insights, we conducted a pilot proof-of-concept study to investigate the diagnostic and prognostic utility of circulating MBG in patients undergoing cardiac surgery, specifically in relation to the risk of a postoperative AKI. By integrating MBG measurements with the STS-AKI score, we sought to determine whether this substance can enhance risk stratification and provide better identification of the patients at prominent risk for this complication.

## 2. Materials and Methods

### 2.1. Study Design

One hundred and ninety-eight adult patients consecutively admitted to the Cardiac Surgery Unit of the Dulbecco University Hospital of Catanzaro (Italy) for cardiac surgery were screened to participate in this pilot, prospective, observational study. The exclusion criteria were emergency cardiac surgery; severely compromised cardiac function; overt chronic kidney disease as defined by a glomerular filtration rate (eGFR) < 60 mL/min/1.73 m^2^; acute infectious liver or autoimmune diseases; and treatment with nephrotoxic medications, immunosuppressors, or contrast medium administration two weeks before surgery.

### 2.2. Data Collection and MBG Measurement

Clinical, anthropometric, laboratory, instrumental, and surgical data were recorded for each patient using a standardized case report form. Common biochemical tests were performed according to the standard methods used in the routine clinical laboratory. The blood samples for the MBG measurement were obtained before surgery and at pre-established time points (4 h, 8 h, and 12 h after surgery). The specimens were centrifuged at 1227 g for 15 min at 4 °C, and the aliquots were stored at −80 °C until they were thawed for the batch analysis. All the MBG measurements were done in the same laboratory (CNR-Institute of Clinical Physiology, Reggio Calabria, Italy) with a commercially available ELISA kit (MyBiosource Inc., San Diego, CA, USA) following the manufacturer’s instructions.

### 2.3. Cardiopulmonary Bypass Procedure

The cardiopulmonary bypass (CPB) circuit and perfusion conduits were standardized for all the patients and remained unchanged during the study period.

Heparin was given at a 300 IU/kg dose to achieve a target-activated clotting time longer than 480 s. The systemic temperature was kept between 32 °C and 34 °C. Myocardial protection was always achieved with intermittent antegrade and retrograde hyperkalemic blood cardioplegia. A crystalloid solution was used for priming the CPB filling. The blood was used to prime the CPB circuit to preserve a high hematocrit. The total CPB flow was maintained at 2.6 L·min^−1^·m^−2^. Protamine was administered at the end of the operation to fully reverse the effects of the heparin.

### 2.4. AKI Endpoint and Risk Assessment

A postoperative AKI represented the study endpoint. This was defined according to the Kidney Disease Improving Global Outcomes (KDIGO) Clinical Practice Guidelines for Acute Kidney Injury as an increase in the postoperative serum creatinine ≥ 0.3 mg/dL within 48 h or an increase > 1.5-fold during the 7 days following surgery. The severity of the AKI was also staged according to the same guidelines [13]. The surgical risk for an AKI was computed by the STS-AKI (Society of Thoracic Surgeons—acute kidney injury) score [3,4], a predictive tool that incorporates various patient factors such as age, gender, preoperative creatinine levels, the preoperative glomerular filtration rate (GFR), the presence of congestive heart failure, diabetes, chronic lung disease, and the history of stroke. Surgical factors like bypass time and cross-clamp time are also considered.

### 2.5. Statistical Analysis

All statistical analyses were performed using the STATA (version 18.0; StataCorp LL, College Station, TX, USA), GraphPad Prism (version 9.0.0; GraphPad Software LLC, San Diego, CA, USA), and MedCalc statistical software (version 14.8.1; MedCalc Software bvba, Ostend, Belgium). The data were presented as the mean ± SD, median [IQ range], or frequency percentage, as appropriate. The differences between the groups were determined by the unpaired *t*-test for normally distributed values, the Mann–Whitney U test for skewed variables, and the chi-square followed by a Fisher’s exact test for frequency distributions. Pairwise comparisons were made by the Wilcoxon test for non-normally distributed values.

An ANOVA for repeated measures was employed to analyze the statistical variance of MBG across the established time points in the whole study population (p for trend) and a test of the between-subjects effects was used to check the differences in the MBG trends between the subgroups of patients with or without a subsequent AKI.

The Receiver Operating Characteristics (ROC) analyses were employed to test the diagnostic capacity of the single values and absolute delta changes of MBG across the different time measurements with respect to an AKI. The Youden index was also computed to establish the best cut-off values for optimal discrimination.

The MBG values exhibiting a statistically significant discriminatory capacity in the ROC analyses were individually combined with the STS-AKI score to assess possible improvements in the overall discrimination (ROC-AUCs), calibration (the Hosmer–Lemeshow test), and explained variance (Nagelkerke R^2^) of this model. Additionally, reclassification analyses were made by assessing the integrated discrimination improvement (IDI) and the net reclassification improvement (NRI) with the patients being arranged according to strata of predicted probability adopting a 0.3 cut-off value. All the results were considered significant for *p* values < 0.05.

## 3. Results

### 3.1. Study Population Characteristics

**Figure 1:**
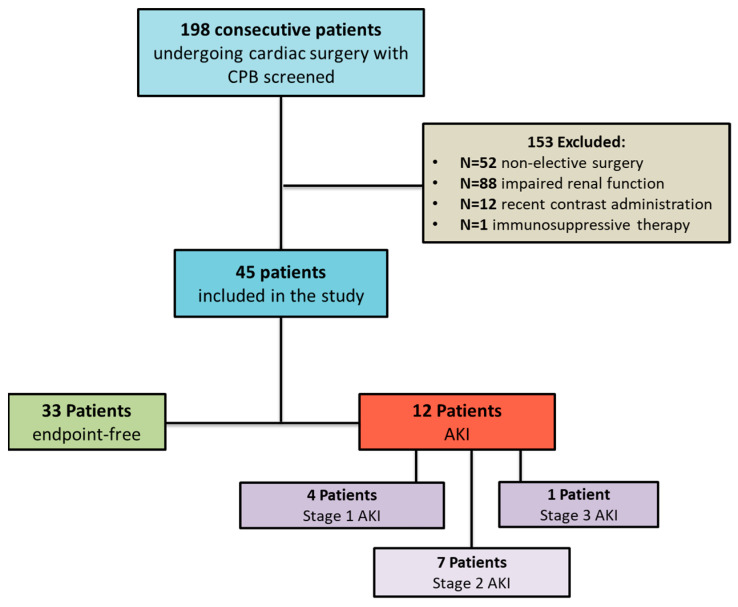
Study population flow.

Forty-five patients matched the inclusion criteria to participate in this study. The main reasons for the non-eligibility of excluded patients were non-elective cardiac surgery, impaired renal function (eGFR < 60 mL/min/1.73 m^2^), and recent contrast medium administration (Figure 1). The mean age of the patients was 65.6 ± 8 years. Most individuals were male (77.8%) and diabetic (60%) with a median diabetes vintage of 7.5 years [IQR 1–12]. Almost all individuals (95.6%) had previous cardio- or cerebrovascular disease. The renal function was conserved with mean serum creatinine values of 0.90 ± 0.19 mg/dL and a mean estimated GFR (CKD-EPI) of 91.8 ± 14.3 mL/min/1.73 m^2^. On average, the patients displayed a preserved ejection fraction (median 50%, IQR 45–55) and a normal/mildly abnormal left atrial volume (42.3 ± 8.27 mL/m^2^). Nearly all patients were on RAS blockers (91.1%), beta-blockers (82.2%), and statins (84.4%). The median STS-AKI score was 1.27 [IQR 0.75–1.99], which indicated, on average, a low propensity for a postoperative AKI occurrence in this cohort. The most frequent surgical intervention was isolated CABG (71.1%), followed by CABG plus valve surgery (15.6%), valve surgery only (11.1%), or other (2.2%). All interventions were successful and well tolerated in all patients with no relevant complications or side events reported. The median cross-clamp time was 72 [IQR 56–104.2] mins while the median CPB duration was 105 [IQR 91–137] mins. Table 1 summarizes the main clinical, anthropometric, laboratory, instrumental, and surgical data of the study population.

### 3.2. MBG Trend and AKI after Cardiac Surgery

**Figure 2:**
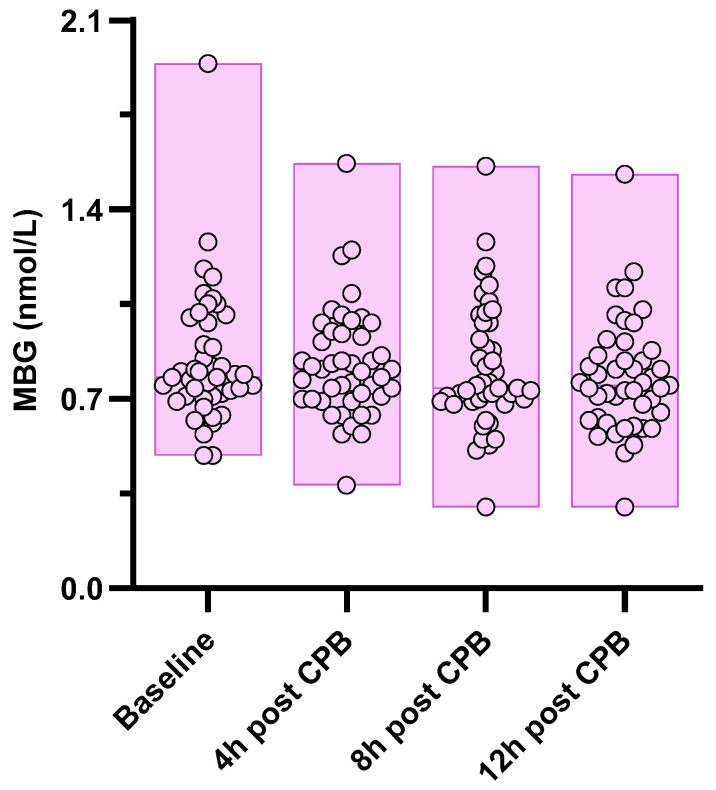
The trend of circulating MBG (individual values + range) after CPB in the whole study population. *p* for trend = 0.02.

The MBG values displayed, on average, a decreasing trend from the baseline to 12 h after surgery (0.812 [IQR 0.712–0.988] to 0.752 [IQR 0.630–0.865] nmol/L; *p* for trend = 0.02; Figure 2).

A post-surgery AKI occurred in 12 patients (26.7%). Of these, four patients had a stage 1 AKI (33.3%), seven had a stage 2 AKI (58.3%) and one patient experienced a stage 3 AKI (8.3%). At the baseline, the AKI patients exhibited significantly lower hemoglobin levels (*p* = 0.04), an increased left atrial volume (*p* = 0.04), a worsened STS-AKI score (*p* = 0.04), and higher CPB and cross-clamp times, compared to others (*p* = 0.002 and 0.0003, respectively). No statistical differences in the other clinical parameters were reported (Table 1).

**Figure 3:**
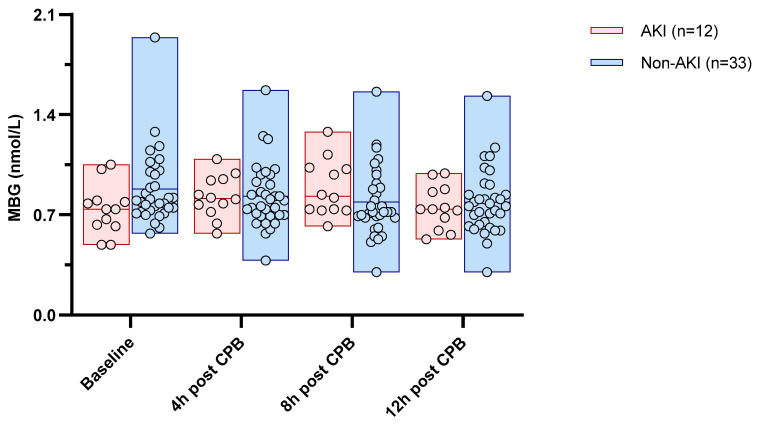
Separate trends of circulating MBG (individual values + range) after CPB in individuals with or without a subsequent AKI. *p* for the trend in the AKI and non-AKI subgroups were 0.26 and 0.005, respectively.

The baseline MBG levels were apparently lower in the patients with a subsequent AKI compared to others (0.738 [0.623–0.795] vs. 0.807 [0.720–1.006] nmol/L), but this difference did not attain statistical significance (*p* = 0.06). The MBG decreased from the baseline to 12 h after surgery in non-AKI individuals (0.807 [0.720–1.006] to 0.763 [0.628–0.873] nmol/L; *p* for trend = 0.005), while no linear trend was observed in the AKI individuals (*p* for trend = 0.26). However, in this latter group, the MBG significantly increased from the baseline to 4 h (0.738 [0.623–0.795] to 0.816 [0.730–0.946]; *p* = 0.03) and even more to 8 h (0.830 [0.730–1.029]; *p* = 0.001) but subsequently decreased at 12 h (0.744 [0.608–0.875]; *p* = 0.001). Figure 3 summarizes the separate trends of circulating MBG in the AKI and non-AKI subgroups.

### 3.3. Diagnostic Performance of MBG in Identifying AKI

The ROC analyses demonstrated a marginal diagnostic performance of the single MBG time values in detecting individuals with a subsequent AKI (Table 2). In more detail, the MBG measured at either 4 h or 12 h after surgery was not statistically discriminant in such regard (AUC: 0.535 [0.381–0.685]; *p* = 0.71 and 0.545 [0.390–0.694], *p* = 0.64, respectively), while a barely significant capacity was observed for the measurements at the baseline (AUC: 0.688 [0.533–0.818]; *p* = 0.04) and 8 h after the CPB (AUC: 0.674 [0.518–0.806; *p* = 0.04)]. On the other hand, all the delta changes displayed a remarkable diagnostic ability to identify AKI patients, except for the 4–12 h (AUC: 0.564 [0.409–0.711]; *p* = 0.49) and the 4–8 h after the CPB (AUC: 0.679 [0.524–0.810]; *p* = 0.05).

In particular, an MBG increase from the baseline to 8 h after the CPB > 0.03 nmol/L held a 91.7% [95% CI; 61.5–99.8] sensitivity and an 81.8% [95% CI; 64.5–93.0] specificity to discriminate AKI patients (AUC: 0.917 [0.795–0.978]; *p* < 0.001), while a decrease from 8 to 12 h after a CPB ≤ 0.06 nmol/L showed a 91.7% [61.5–99.8] sensitivity and a 69.7% [51.3–84.4] specificity with an AUC of 0.843 [0.704–0.934] (*p* < 0.001). Table 3 provides the detailed data on the ROC analyses for all the delta changes in MBG across the various time measurements.

### 3.4. Incremental Value of MBG on the STS-AKI Score Performance

In this cohort, the STS-AKI score displayed a good diagnostic capacity to identify AKI patients (AUC: 0.736 [95% CI; 0.581–0.857]; *p* = 0.006). This risk model was quite well calibrated (X^2^ = 7.89; *p* = 0.57) but limited in explaining the overall risk variance in this cohort (R^2^ = 0.17; *p* = 0.02).

The inclusion of the baseline or 8 h post-CPB MBG values increased the discriminatory capacity of this model (AUC: 0.771 [95% CI; 0.620–0.884]; *p* = 0.002 and 0.810 [0.663–0.912], and *p* < 0.001, respectively) and slightly improved the explained variance and the overall calibration but did not modify in a significant manner the model reclassification capacity (NRI and IDI).

By the same token, the addition of delta MBG changes from the baseline to 4 h, the baseline to 12 h, and 4 h to 8 h augmented the diagnostic capacity (AUCs ranging from 0.785 to 0.816; all *p* < 0.001), the explained variance, and the overall calibration of the STS-AKI score but had uncertain effects on the reclassification capacity. Conversely, the MBG changes from the baseline to 8 h and from 8 h to 12 h post-CPB exhibited a remarkable impact on all performance domains, markedly increasing the discrimination capacity (AUCs: 0.949 [0.840–0.993] and 0.904 [0.779–0.971], respectively; *p* < 0.001 for both), the calibration (X^2^ 6.89, *p* = 0.44 and 7.87, *p* = 0.24, respectively) and, to a more significant extent, the explained variance (0.69 and 0.56, respectively; both *p* < 0.001), as well as the reclassification capacity (NRI +36.3%; *p* = 0.04 for both; and IDI +45.9% and +35.7%, respectively; both *p* < 0.001). Table 4 summarizes the STS-AKI model performance before and after the inclusion of the MBG measurement.

## 4. Discussion

An AKI remains a significant concern following cardiac surgery, posing a substantial risk to patient outcomes. Indeed, although preliminary, our findings suggest that the MBG measurement may hold usefulness for the AKI risk stratification and, to a more significant extent, for improving the predictive capacity of a widely employed risk prediction model, namely, the STS-AKI score.

Interestingly, such a score was overall low in our study cohort (median 1.27; IQR 0.75–1.99), a finding which pairs well with the conserved preoperative renal and cardiac function of most patients and the elective nature of all surgeries.

Yet, an AKI occurred in twelve (26.7%) patients with the majority experiencing moderate to severe stages, a figure which is in line with the general incidence of this complication in similar risk settings [1,14]. The patients who developed an AKI had longer cardiopulmonary bypass (CPB) and cross-clamp times, lower hemoglobin levels, and increased left atrial volumes at the baseline. These findings are consistent with several previous observations, emphasizing the multifactorial nature of an AKI, where prolonged surgical times and hemodynamic instability play critical roles [15].

Of note, the diagnostic performance of the STS-AKI score, even in such a low-risk cohort, was rather satisfactory, yielding a ROC-AUC of 0.736; however, despite also being well calibrated, this model had limited value in explaining the overall AKI risk variability in this cohort (17%).

Nonetheless, a significant aspect of our study was to analyze the trend in circulating MBG levels throughout the perioperative period. The MBG levels generally decreased from baseline to 12 h post-surgery in the overall cohort. However, a different pattern emerged in the patients who developed an AKI, where the MBG levels initially rose at 4 h post-surgery, peaked at 8 h, and then decreased by 12 h. This biphasic response suggests a possible compensatory response following the ischemic and inflammatory stress experienced by the kidneys [9]. Additionally, the hyperactivation of the renin–angiotensin system (RAS) following prolonged renal ischemia cannot be ruled out, considering that under either physiological or pathological conditions peripheral RAS activation remains one of the stronger triggers for MBG release [16]. Given the observational nature of our study, we cannot further clarify this issue. Future targeted studies are desirable to throw light on these observations from a mechanistic perspective.

Nevertheless, to our opinion, the key aspect of our study pertains to the possible clinical significance of MBG measurement as a stand-alone and, even more, as a complementary biomarker to the STS-AKI score for refining the AKI risk assessment.

Of note, in evaluating the diagnostic potential of MBG, we found that only the measurements at the baseline and 8 h post-CPB held a significant although limited discriminative capacity (AUCs: 0.688 and 0.674, respectively). Conversely, like the frequently reported other biomarkers [17], the changes in MBG over time provided more significant insights. An increase in the MBG from the baseline to 8 h post-CPB (AUC: 0.917), as well as the following reduction from 8 to 12 h CPB (AUC: 0.843), were particularly telling in such regard, displaying a very high accuracy for AKI detection. This dynamic change in MBG supports the hypothesis that this biomarker may reflect acute renal stress and damage more accurately than static measurements. The initial rise in the MBG could indicate a response to ischemic injury or other perioperative stresses, while the subsequent decline might reflect recovery or adaptation mechanisms.

As alluded to before, the STS-AKI score alone showed a good predictive capacity for an AKI in our patients, which is an expected find given its design and widespread use. However, when we integrated the MBG measurements into this model, its overall performance improved significantly. This, again, was more evident considering the delta MBG changes, rather than single time point measurements.

In particular, the discrimination capacity of the STS-AKI score—that is, the model’s ability to correctly distinguish between patients who will and will not develop an AKI—rose from 0.736 to 0.949 and 0.904, respectively, after including the data on the MBG changes from the baseline to 8 h and from 8 to 12 h post-CPB. Importantly, these two delta changes were the only ones able to improve all the other model performance domains in a significant manner. Model calibration, for instance, is a critical aspect of multivariable prognostic tools as it assesses how closely the predicted probabilities of an outcome match the actual occurrence [18]. The improved calibration of the STS-AKI score following the inclusion of MBG, particularly the two above-mentioned delta changes, means that the predicted risk of an AKI more accurately reflects the true risk observed in this population. This adjustment may help ensure that the model’s risk estimates are reliable and meaningful in clinical practice, although this observation requires appropriate confirmation in larger external cohorts.

As mentioned earlier, one strong limitation of the STS-AKI score in this cohort was the capacity to only account for a minor proportion (17%) of the AKI variability. As for the other parameters considered, the explained variance of this score was also remarkably increased by the inclusion of the MBG changes (from 17 to 69 and 56%, respectively, with delta baseline–8 h and delta 8–12 h in the model); this indicates that this refined risk model better captures the factors contributing to an AKI, thus providing a more comprehensive risk assessment [19].

Finally, and no less importantly, the integration of dynamic MBG measurements with the STS-AKI score also improved to a substantial extent the model reclassification capacity. Reclassification evaluates whether the inclusion of a new biomarker, like MBG, correctly changes the risk categories of patients [18,20]. Improved reclassification means that MBG helps more accurately assign patients to appropriate risk categories, enhancing clinical decision-making. Our study showed significant changes in reclassification, with net reclassification improvement (NRI) and integrated discrimination improvement (IDI) statistics indicating remarkable gains.

Taken together, these advancements suggest that MBG can provide crucial insights into individual patient risk, leading to more tailored and effective interventions.

Although interesting, our study has some limitations that relate to the pilot and exploratory nature of our investigation. First, the small sample size and the low number of AKI events collected, despite being in line with the overall incidence of this complication, prevented us from performing stratified analyses on the patients’ characteristics, severity of the AKI, and long-term outcomes. By the same token, we only included according to the protocol the patients undergoing elective surgery and with conserved preoperative renal function. This aspect could raise concerns about possible selection bias, hampering the overall applicability of the findings in more heterogeneous and high-risk cohorts. Third, the limited number of MBG time measurements could not fully capture the overall trend of this substance in the whole cohort and in separate subgroups; more in-depth investigations are advocated to identify more alternative MBG cut-points with a better capacity of risk stratification.

## 5. Conclusions

Our study suggests that the MBG measurement can significantly enhance the prediction of an AKI in cardiac surgery patients. The integration of dynamic MBG changes with the STS-AKI score offers a more nuanced and accurate risk stratification, which could lead to earlier identification and intervention for at-risk patients; hence, this study gives another example of the importance of integrating novel biomarkers with existing clinical models to enhance patient care and outcomes.

Future research should aim to validate these findings in larger and miscellaneous patient populations and to understand the underlying biological mechanisms linking MBG to renal injury. Additionally, exploring MBG-guided therapeutic strategies could help tailor perioperative care to individual patient needs, potentially reducing the incidence and severity of an AKI.

## Figures and Tables

**Table 1:** Main characteristics of the study cohort and differences between subgroups of patients with or without a subsequent AKI. Statistically significant differences are highlighted in bold.

	All *n* = 45	AKI *n* = 12	Non-AKI *n* = 33	*p*
**Patients’ characteristics**				
Age (years)	65.6 ± 8	64.1 ± 7.9	64.9 ± 8	0.93
Gender (%Male)	77.8	75	78.8	0.78
BMI (kg/m)^2^	27.8 [25.9–30.9]	32.5 ± 8.2	27.8 [25.9–29.8]	0.16
Smoking (%)	35.5	16.7	42.4	0.52
CV disease (%)	95.6	91.7	97	0.98
Hypertension (%)	73.3	66.7	75.7	0.39
Diabetes (%)	60	66.7	57.6	0.73
Diabetes vintage (yrs)	7.5 [1–12]	7 [3–11.5]	7.5 [1–12]	0.89
NYHA class (%1/2/3)	15.6/64.4/20	0/66.7/33.3	21.2/63.6/15.2	0.55–0.82
Ejection fraction (%)	50 [45–55]	50.4 ± 6.1	49.6 ± 9	0.69
**Left atrial volume (mL/m^2^)**	**42.3 ± 8.27**	**46.3 ± 6.7**	**40.4 ± 8.4**	**0.04**
Creatinine (mg/dL)	0.90 ± 0.19	0.93 ± 0.20	0.90 ± 0.20	0.81
eGFR CKD-EPI (mL/min/m^2^)	91.8 ± 14.3	91 ± 14.7	92.1 ± 14.3	0.77
Urea (mg/dL)	39 [31.7–47.2]	42.5 [32.5–52.5]	39 [31.7–46.2]	0.21
**Haemoglobin (g/dL)**	**12.8 ± 1.5**	**12.6 ± 1.5**	**13.8 ± 1.6**	**0.04**
Haematocrit (%)	38.7 ± 4.7	40.4 ± 5.8	38.1 ± 4.2	0.36
Total cholesterol (mg/dL)	142.7 ± 43.1	145.7 ± 33.5	141.6 ± 46.7	0.78
LDL cholesterol (mg/dL)	80 [62.2–108.7]	88.9 ± 28.3	80 [60.5–112.2]	0.89
Triglycerides (mg/dL)	102 [86–131]	97.5 [79.5–126.5]	104 [86–132]	0.75
CK-MB (UI/L)	1.6 [1.3–2.4]	1.85 [1.50–2.20]	1.6 [1.2–2.42]	0.92
Hs-cTN (ng/L)	16 [9.3–26.6]	15.8 [13.2–25.6]	16.6 [9.1–28.8]	0.85
Myoglobin (ng/mL)	29 [22.5–45.7]	29.5 [26.5–37.5]	28 [21–48]	0.77
**Preoperative medications**				
ACEi/ARBs (%)	91.1	83.3	90.9	0.66
Diuretics (%)	44.4	41.6	45.4	0.70
Beta-blockers (%)	82.2	83.3	81.8	1.00
Calcium channel blockers (%)	15.5	16.7	15.1	0.84
Statins (%)	84.4	83.3	84.5	0.92
Platelet inhibitors (%)	28.9	25	30.3	0.89
**Surgical characteristics**				
Type of surgery				
CABG only (%)	71.1	41.7	81.8	0.33
CABG plus valve (%)	15.6	25	12.1	0.45
Valve only (%)	11.1	25	6.1	0.29
Other (%)	2.2	8.3	0	0.58
Preoperative SBP (mmHg)	130.1 ± 15.7	129 [120.5–132.5]	130.6 ± 16.2	0.76
Preoperative DBP (mmHg)	74.6 ± 10.9	74.6 ± 11.3	74.6 ± 10.9	0.91
**STS-AKI score**	**1.27 [0.75–1.99]**	**3.09 ± 1.12**	**1.08 [0.58–1.62]**	**0.04**
**Cross-clamp time (min)**	**72 [56–104.2]**	**103.8 ± 33.1**	**69.1 ± 23.5**	**<0.001**
**CPB time (min)**	**105 [91–137]**	**147.6 ± 59.3**	**104.7 ± 30.6**	**0.002**
**MBG measurement**				
Baseline MBG (nmol/L)	0.802 [0.712–0.988]	0.738 [0.623–0.795]	0.807 [0.720–1.006]	0.06
MBG 4 h post-CBP (nmol/L)	0.781 [0.699–0.942]	0.816 [0.730–0.946]	0.802 [0.695–0.956]	0.73
MBG 8 h post-CBP (nmol/L)	0.738 [0.692–0.979]	0.830 [0.730–1.029]	0.722 [0.684–0.902]	0.08
MBG 12 h post-CBP (nmol/L)	0.752 [0.630–0.865]	0.744 [0.608–0.875]	0.763 [0.628–0.873]	0.66

Legend: AKI: acute kidney injury; BMI: body mass index; CABG: coronary artery bypass graft; CK-MB: creatine kinase MB; CPB: cardiopulmonary bypass; CV: cardiovascular; DBP: diastolic blood pressure; eGFR: estimated glomerular filtration rate; Hs-cTN: highly sensitive c-troponin; LDL: low-density lipoprotein; NYHA: New York Health Association; MBG: marinobufagenin; SBP: systolic blood pressure; and STS: Society of Thoracic Surgeons.

**Table 2:** Areas under the curve (AUCs) and best cut-off values (Youden index) of the circulating MBG were measured at different time points to detect patients with a subsequent AKI. Statistically significant AUCs are highlighted in bold.

MBG Time Measurement	AUC [95% CI]	*p*	Best Cut-Off (nmol/L)	Sens.% [95% CI]	Spec.% [95% CI]
**Baseline MBG**	**0.688 [0.533–0.818]**	**0.04**	**≤0.669**	**41.6 [15.2–72.3]**	**90.9 [75.7–98.1]**
MBG 4 h post-CPB	0.535 [0.381–0.685]	0.71	>0.757	75.0 [42.8–94.5]	48.4 [30.8–66.5]
**MBG 8 h post-CPB**	**0.674 [0.518–0.806]**	**0.04**	**>0.724**	**91.7 [61.5–99.8]**	**54.5 [36.4–71.9]**
MBG 12 h post-CPB	0.545 [0.390–0.694]	0.64	≤0.669	66.7 [34.9–90.1]	54.5 [36.4–71.9]

Legend: AUC: area under the curve; CPB: cardiopulmonary bypass; MBG: marinobufagenin; Sens.: sensitivity; and Spec.: specificity.

**Table 3:** Areas under the curve (AUCs) and best cut-off values (Youden index) of the delta changes in the circulating MBG to detect patients with a subsequent AKI. Statistically significant AUCs are highlighted in bold.

MBG Change	AUC [95% CI]	p	Best Cut-Off (Absolute Change)	Sens.% [95% CI]	Spec.% [95% CI]
**Δ baseline–4 h post-CPB**	**0.707 [0.553–0.833]**	**0.008**	**>−0.06**	**91.7 [61.5–99.8]**	**57.6 [39.2–74.5]**
**Δ baseline–8 h post-CPB**	**0.917 [0.795–0.978]**	**<0.001**	**>0.03**	**91.7 [61.5–99.8]**	**81.8 [64.5–93.0]**
**Δ baseline–12 h post-CPB**	**0.737 [0.585–0.857]**	**0.001**	**>−0.08**	**100 [73.5–100.0]**	**60.6 [42.1–77.1]**
**Δ 4–8 h post-CPB**	**0.679 [0.524–0.810]**	**0.05**	**>0.011**	**75.0 [42.8–94.5]**	**69.7 [51.3–84.4]**
Δ 4–12 h post-CPB	0.564 [0.409–0.711]	0.49	≤0.107	100 [73.5–100]	21.2 [9.0–38.9]
**Δ 8–12 h post-CPB**	**0.843 [0.704–0.934]**	**<0.001**	**≤−0.06**	**91.7 [61.5–99.8]**	**69.7 [51.3–84.4]**

Legend: AUC: area under the curve; CPB: cardiopulmonary bypass; MBG: marinobufagenin; Sens.: sensitivity; and Spec.: specificity.

**Table 4:** Changes in the STS-AKI score performance after inclusion of the different ROC discriminant MBG values (as indicated in Table 3). MBG values imparting a significant improvement in all performance domains are highlighted in bold.

Model	Discrimination	Calibration	Explained Variance		Reclassification	
	AUC [95% CI]	*p*	Hosmer-Lemeshow X^2^	Nagelkerke R^2^	NRI (%) *	IDI (%)
STS-AKI score alone	0.736 [0.581–0.857]	0.006	7.89 (*p* = 0.57)	0.17 (*p* = 0.02)	-	-
**+ single time MBG**						
Baseline MBG	0.771 [0.620–0.884]	0.002	6.36 (*p* = 0.70)	0.27 (*p* = 0.01)	+10.6 (*p* = 0.56)	+10.1 (*p* = 0.10)
MBG 8 h post-CPB	0.810 [0.663–0.912]	<0.001	9.38 (*p* = 0.40)	0.23 (*p* = 0.02)	+5.3 (*p* = 0.59)	+3.1 (*p* = 0.25)
**+ delta changes in MBG**						
Δ baseline–4 h post-CPB	0.816 [0.672–0.915]	<0.001	9.52 (*p* = 0.21)	0.30 (*p* = 0.005)	+18.9 (*p* = 0.24)	+11.0 (*p* = 0.09)
**Δ baseline–8 h post-CPB**	**0.949** **[0.840–0.993]**	**<0.001**	**6.89 (*p* = 0.44)**	**0.69 (*p* < 0.001)**	**+36.3 (*p* = 0.04)**	**+45.9 (*p* < 0.001)**
Δ baseline–12 h post-CPB	0.801 [0.655–0.905]	<0.001	10.03 (*p* = 0.21)	0.28 (*p* = 0.007)	+10.6 (*p* = 0.44)	+9.5 (*p* = 0.11)
Δ 4–8 h post-CPB	0.785 [0.638–0.894]	<0.001	7.94 (*p* = 0.34)	0.23 (*p* = 0.02)	−3.0 (*p* = 0.82)	+5.6 (*p* = 0.13)
**Δ 8–12 h post-CPB**	**0.904** **[0.779–0.971]**	**<0.001**	**7.87 (*p* = 0.24)**	**0.56 (*p* < 0.001)**	**+36.3 (*p* = 0.04)**	**+35.7 (*p* < 0.001)**

Legend: AUC: area under the curve; CPB: cardiopulmonary bypass; IDI: integrated discrimination improvement; MBG: marinobufagenin; NRI: net reclassification improvement; and STS-AKI: Society of Thoracic Surgeons—acute kidney injury. * Patient strata were arranged according to a cut-off value of the predicted probability of 0.3.

## Data Availability

Raw data are available from the corresponding author upon reasonable request.
